# Research on Radiation Characteristic of Plasma Antenna through FDTD Method

**DOI:** 10.1155/2014/290148

**Published:** 2014-07-09

**Authors:** Jianming Zhou, Jingjing Fang, Qiuyuan Lu, Fan Liu

**Affiliations:** School of Information and Electronics, Beijing Institute of Technology, Beijing 100081, China

## Abstract

The radiation characteristic of plasma antenna is investigated by using the finite-difference time-domain (FDTD) approach in this paper. Through using FDTD method, we study the propagation of electromagnetic wave in free space in stretched coordinate. And the iterative equations of Maxwell equation are derived. In order to validate the correctness of this method, we simulate the process of electromagnetic wave propagating in free space. Results show that electromagnetic wave spreads out around the signal source and can be absorbed by the perfectly matched layer (PML). Otherwise, we study the propagation of electromagnetic wave in plasma by using the Boltzmann-Maxwell theory. In order to verify this theory, the whole process of electromagnetic wave propagating in plasma under one-dimension case is simulated. Results show that Boltzmann-Maxwell theory can be used to explain the phenomenon of electromagnetic wave propagating in plasma. Finally, the two-dimensional simulation model of plasma antenna is established under the cylindrical coordinate. And the near-field and far-field radiation pattern of plasma antenna are obtained. The experiments show that the variation of electron density can introduce the change of radiation characteristic.

## 1. Introduction 

Plasma antenna usually adopts the partially or fully ionized gas as conducting medium instead of metallic materials. Compared with conventional metallic antenna, plasma antenna has many peculiar properties. For instance, it can be rapidly switched on or off; this characteristic makes plasma antenna suitable for stealth applications for military communication fields. Also, if this kind of antenna is used as the antenna array, the coupling between the elements of antenna array is small. In particular, radiation pattern of plasma antenna can be reconfigured through changing the frequency and intensity of pump signal, gas pressure, vessel dimensions, and so on. Because of the advantages above, many researchers and scientific utilities show great interests in it.

At present, studies concerning plasma antenna may have three aspects: experimental investigation, theory derivation, and numerical calculation. Theodore Anderson together with Igor Alexeff [1] designed a smart plasma antenna and implemented a wide range of plasma antenna experiments. Their studies had proved that plasma antenna has reconfigurable characteristics. Kumar and Bora [2] designed a 30 cm plasma antenna and proved that the frequency and radiation pattern can be altered with the frequency and power of the pump signal. Yang et al. [3] and Zhao [4] obtained the dispersion relationships of the surface wave along the plasma column by using theoretical derivation approach. Wu et al. [5] and Xia and Yin [6] studied the radiation characteristic of plasma antenna through theoretical derivation. Dai et al. [7] calculated the coefficients of reflection and transmission of electromagnetic wave in plasma by using FDTD numerical method. Liang [8] simulated the radiation characteristic of cylindrical monopolar antenna by using FDTD method. Russo et al. [9–13] established one-dimensional and two-dimensional self-consistent model of plasma antenna and validated the correctness of the model through using FDTD method.

From the investigations and research mentioned above, we can draw a conclusion that plasma is so complicated that one cannot find the real issues of the problem only through experimental approach. It is necessary to establish a rigorous mathematical model to investigate the radiation characteristic of plasma antenna. The numerical calculation approach applied in this paper is to study the radiation characteristic of plasma antenna.

## 2. Propagation of Electromagnetic Wave in Free Space and Plasma

There are two key issues to deal with in this research: one is the propagation of electromagnetic wave in free space and the other one is the propagation of electromagnetic wave in the plasma. Only these two problems are solved; then the investigation of radiation characteristic of plasma antenna can be further conducted.

### 2.1. Propagation of Electromagnetic Wave in Free Space

In order to apply FDTD method to simulate the propagation of electromagnetic wave in free space in cylindrical coordinate, the stretched coordinate is selected. So, the modified Maxwell equations can be expressed as below:

(1) $$\begin{array}{c} \nabla_{s}\times H=j\omega \varepsilon E, \end{array}$$

(2) $$\begin{array}{c} \nabla_{s}\times E=-j\omega \mu H, \end{array}$$

where **E** represents electric field strength vector in volts per meter. **H** represents magnetic field strength vector in amperes per meter. *ε* denotes the permittivity in farad per meter. *μ* denotes the permeability in henry per meter. *ω* represents the angular frequency of incidence signal in radian per second.

In stretched coordinate [14], we define

(3) $$\begin{array}{c} s_{r}=1+\frac{\sigma_{r}}{j\omega \varepsilon_{0}},\\ s_{z}=1+\frac{\sigma_{z}}{j\omega \varepsilon_{0}}, \end{array}$$

where *s*_*r*_ and *s*_*z*_ are coordinate stretched factor

(4) R⟶∫0rsr(r′)dr′ ={r,r′<r0r+1jωε0∫r0rσr(r′)dr′,r′<r0,

(5) Z⟶∫0zsz(z′)dz′ ={z,z′<z0z+1jωε0∫z0zσz(z′)dz′,z′<z0,

where *r*_0_ and *z*_0_ represent the distance between the signal source and inner boundary of PML along *r* direction and *z* direction, respectively.

Maxwell curl equation (1), then, can be represented by these three scale equations in cylindrical coordinate system as (6a)–(6c):

(6a) $$\begin{array}{c} j\omega \varepsilon_{0}E_{r}=\frac{1}{R}\frac{\partial H_{z}}{\partial \varphi }-\frac{\partial H_{\varphi }}{\partial Z}, \end{array}$$

(6b) $$\begin{array}{c} j\omega \varepsilon_{0}E_{\varphi }=\frac{\partial H_{r}}{\partial Z}-\frac{\partial H_{z}}{\partial R}, \end{array}$$

(6c) $$\begin{array}{c} j\omega \varepsilon_{0}E_{z}=\frac{1}{R}\frac{\partial \left(RH_{\varphi }\right)}{\partial R}-\frac{1}{R}\frac{\partial H_{r}}{\partial \varphi }. \end{array}$$

From (4) and (5), (7) can be obtained as follows:

(7) $$\begin{array}{c} \frac{\partial }{\partial R}=\frac{1}{s_{r}}\frac{\partial }{\partial r},\;\;\frac{\partial }{\partial Z}=\frac{1}{s_{z}}\frac{\partial }{\partial z}. \end{array}$$

Substituting (7) into (6a)–(6c) yields

(8a) $$\begin{array}{c} j\omega \varepsilon_{0}E_{r}=\frac{1}{R}\frac{\partial H_{z}}{\partial \varphi }-\frac{1}{s_{z}}\frac{\partial H_{\varphi }}{\partial z}, \end{array}$$

(8b) $$\begin{array}{c} j\omega \varepsilon_{0}E_{\varphi }=\frac{1}{s_{z}}\frac{\partial H_{r}}{\partial z}-\frac{1}{s_{r}}\frac{\partial H_{z}}{\partial r}, \end{array}$$

(8c) $$\begin{array}{c} j\omega \varepsilon_{0}E_{z}=\frac{1}{R}\frac{1}{s_{r}}\frac{\partial \left(RH_{\varphi }\right)}{\partial r}-\frac{1}{R}\frac{\partial H_{r}}{\partial \varphi }. \end{array}$$

After multiplying *s*_*z*_ · *R*/*r*, *s*_*z*_ · *s*_*r*_, *s*_*r*_ · *R*/*r*, respectively, (8a), (8b), and (8c) can be expressed as below:

(9a) $$\begin{array}{c} j\omega \varepsilon_{0}s_{z}\frac{R}{r}E_{r}=\frac{1}{r}\frac{\partial \left(s_{z}H_{z}\right)}{\partial \varphi }-\frac{R}{r}\frac{\partial H_{\varphi }}{\partial z}, \end{array}$$

(9b) $$\begin{array}{c} j\omega \varepsilon_{0}s_{z}s_{r}E_{\varphi }=\frac{\partial \left(s_{r}H_{r}\right)}{\partial z}-\frac{\partial \left(s_{z}H_{z}\right)}{\partial r}, \end{array}$$

(9c) $$\begin{array}{c} j\omega \varepsilon_{0}s_{r}\frac{R}{r}E_{z}=\frac{1}{r}\frac{\partial \left(RH_{\varphi }\right)}{\partial r}-\frac{1}{r}\frac{\partial \left(s_{r}H_{r}\right)}{\partial \varphi }. \end{array}$$

Substituting *s*_*z*_ *H*_*z*_ = *H*_*z*_′, *s*_*r*_ *H*_*r*_ = *H*_*r*_′, *RH*_*φ*_/*r* = *H*_*φ*_′, *RE*_*φ*_/*r* = *E*_*φ*_′, *s*_*z*_ *E*_*z*_ = *E*_*z*_′, and *s*_*r*_ *E*_*r*_ = *E*_*r*_′ into (9a)–(9c), (9a)–(9c) can be written as

(10a) $$\begin{array}{c} j\omega \varepsilon_{0}\frac{s_{z}}{s_{r}}\frac{R}{r}E_{r}^{\prime }=\frac{1}{r}\frac{\partial H_{z}^{\prime }}{\partial \varphi }-\frac{\partial H_{\varphi }^{\prime }}{\partial z}, \end{array}$$

(10b) $$\begin{array}{c} j\omega \varepsilon_{0}s_{z}s_{r}\frac{r}{R}E_{\varphi }^{\prime }=\frac{\partial \left(H_{r}^{\prime }\right)}{\partial z}-\frac{\partial \left(H_{z}^{\prime }\right)}{\partial r}, \end{array}$$

(10c) $$\begin{array}{c} j\omega \varepsilon_{0}\frac{s_{r}R}{s_{z}r}E_{z}^{\prime }=\frac{1}{r}\frac{\partial \left(rH_{\varphi }^{\prime }\right)}{\partial r}-\frac{1}{r}\frac{\partial \left(H_{r}^{\prime }\right)}{\partial \varphi }. \end{array}$$

Namely,

(11) $$\begin{array}{c} \left[\begin{array}{c} \frac{1}{r}\frac{\partial H_{z}^{\prime }}{\partial \varphi }-\frac{\partial H_{\varphi }^{\prime }}{\partial z}\\ \frac{\partial \left(H_{r}^{\prime }\right)}{\partial z}-\frac{\partial \left(H_{z}^{\prime }\right)}{\partial r}\\ \frac{1}{r}\frac{\partial \left(rH_{\varphi }^{\prime }\right)}{\partial r}-\frac{1}{r}\frac{\partial \left(H_{r}^{\prime }\right)}{\partial \varphi } \end{array}\right]=j\omega \varepsilon_{0}\varepsilon_{r}\overset{-}{\varepsilon }\left[\begin{array}{c} E_{r}^{\prime }\\ E_{\varphi }^{\prime }\\ E_{z}^{\prime } \end{array}\right]. \end{array}$$

Equation (11) can be shortly expressed as

(12) $$\begin{array}{c} \nabla \times H=j\omega \varepsilon_{0}\varepsilon_{r}\overset{-}{\varepsilon }E. \end{array}$$

According to the duality theorem, Maxwell curl equation (2) can be represented by equation

(13) $$\begin{array}{c} \left[\begin{array}{c} \frac{1}{r}\frac{\partial E_{z}^{\prime }}{\partial \varphi }-\frac{\partial E_{\varphi }^{\prime }}{\partial z}\\ \frac{\partial \left(E_{r}^{\prime }\right)}{\partial z}-\frac{\partial \left(E_{z}^{\prime }\right)}{\partial r}\\ \frac{1}{r}\frac{\partial \left(rE_{\varphi }^{\prime }\right)}{\partial r}-\frac{1}{r}\frac{\partial \left(E_{r}^{\prime }\right)}{\partial \varphi } \end{array}\right]=-j\omega \mu_{0}\mu_{r}\overset{-}{\mu }\left[\begin{array}{c} H_{r}^{\prime }\\ H_{\varphi }^{\prime }\\ H_{z}^{\prime } \end{array}\right]. \end{array}$$

Equation (13) can be expressed as

(14) $$\begin{array}{c} \nabla \times E=-j\omega \mu_{0}\mu_{r}\overset{-}{\mu }H, \end{array}$$

where

(15) $$\begin{array}{c} \overset{-}{\varepsilon }=\overset{-}{\mu }=\left[\begin{array}{ccc} \frac{s_{z}R}{s_{r}r} & 0 & 0\\ 0 & \frac{s_{z}s_{r}r}{R} & 0\\ 0 & 0 & \frac{s_{r}R}{s_{z}r} \end{array}\right]. \end{array}$$

AS the plasma antenna is rotationally symmetric. Thus, it is suitable to study this problem in cylindrical coordinate. The TM modes are excited. Maxwell equations involve three components: *E*_*r*_, *E*_*z*_, and *H*_*φ*_. Thus, the Maxwell equation of electromagnetic wave propagating in free space will be reduced as

(16a) $$\begin{array}{c} -j\omega \mu_{0}s_{z}s_{r}\frac{r}{R}H_{\varphi }^{\prime }=\frac{\partial \left(E_{r}^{\prime }\right)}{\partial z}-\frac{\partial \left(E_{z}^{\prime }\right)}{\partial r}, \end{array}$$

(16b) $$\begin{array}{c} j\omega \varepsilon_{0}\frac{s_{z}}{s_{r}}\frac{R}{r}E_{r}^{\prime }=-\frac{\partial H_{\varphi }^{\prime }}{\partial z}, \end{array}$$

(16c) $$\begin{array}{c} j\omega \varepsilon_{0}\frac{s_{r}R}{s_{z}r}E_{z}^{\prime }=\frac{1}{r}\frac{\partial \left(rH_{\varphi }^{\prime }\right)}{\partial r}. \end{array}$$

Applying the auxiliary differential equation method (ADE) [15], the iterative equations [16] of (16a)–(16c) are derived as follows:

(17a) Bφ ∣ i,jn+1=(2ε0−dtσr2ε0+dtσr)Bφ ∣ i,jn +(2ε0dt2ε0+dtσr)[Ez ∣ i+1/2,jn+1/2−Ez ∣ i−1/2,jn+1/2dr−Er ∣ i,j+1/2n+1/2−Er ∣ i,j−1/2n+1/2dz],

(17b) Hφ ∣ i,jn+1=(2ε0−σzdt2ε0+σzdt)Hφ ∣ i,jn+1 +2ε0R(2ε0+σzdt)μ0μrr(Bφ ∣ i,jn+1−Bφ|i,jn),

(18a) Dr ∣ i+1/2,j,kn+1=(2ε0−σzdt2ε0+σzdt)Dr ∣ i+1/2,j,kn+(2ε0dt2ε0+σzdt) ×{1ri+1/2Hz ∣ i+1/2,j+1/2,kn+1/2−Hz ∣ i+1/2,j−1/2.kn+1/2dφ−Hφ ∣ i+1/2,j,k+1/2n+1/2−Hφ ∣ i+1/2,j·k−1/2n+1/2dz},

(18b) Er ∣ i+1/2,j,kn+1=Er ∣ i+1/2,j,kn +rε0εrR(2ε0+dtσr2ε0Dr ∣ i+1/2,j,kn+1−2ε0−dtσr2ε0Dr ∣ i+1/2,j,kn),

(19a) Dz ∣ i,j,k+1/2n+1=(2ε0−σrdt2ε0+σrdt)Dz ∣ i,j,k+1/2n+(2ε0dt2ε0+σrdt) ×{(12r+1dr)Hφ ∣ i+1/2,j,k+1/2n+1/2+(12r−1dr)Hφ ∣ i−1/2,j,k+1/2n+1/2,},

(19b) Ez ∣ i,j,k+1/2n+1=Ez ∣ i,j,k+1/2n +rε0εrR(2ε0+dtσz2ε0Dz ∣ i,j,k+1/2n+1−2ε0−dtσz2ε0Dz ∣ i,j,k+1/2n).

By using these six iterative equations, we can calculate the value of electromagnetic field in PML. Also, we can use these iterative equations to calculate the value of electromagnetic field in free space by setting the electric conductivity at *σ*_*z*_ = 0, *σ*_*r*_ = 0, and *ε*_*r*_ = 1.

**Figure 1:**
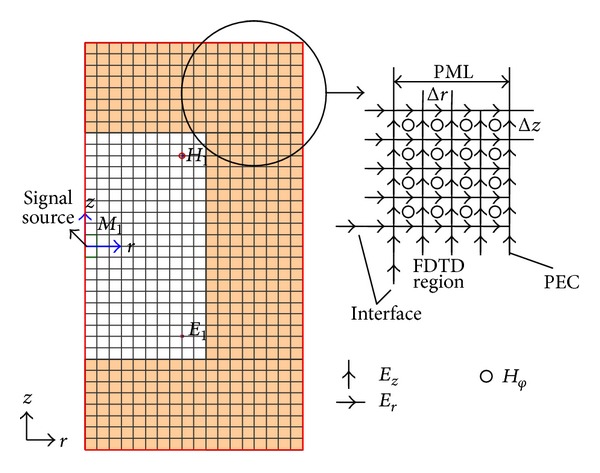
Two-dimensional FDTD computational space.

In order to validate the correctness of the theory above, we apply this approach in the propagation of electromagnetic field in free space. The two-dimensional FDTD computational space is shown as in Figure 1.

**Figure 2:**
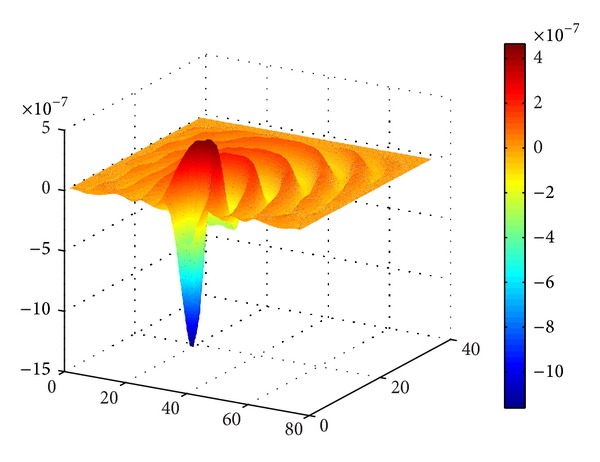
Propagating the electric field *E*_*r*_ in free space.

Figure 1 shows that half of the free space is simulated. The computational space is composed of 50 × 100 Yee sells. The signal source is sinusoidal signal with the frequency of 20 GHz. The spatial step is Δ*r* = Δ*z* = 0.003 m. The temporal step is Δ*t* = 2.123 × 10^−12^ s. The total number of time steps is 500. The number of PML cells is 9. The propagating process of electric field *E*_*r*_ in free space is shown as in Figure 2.

In Figure 2, it is shown that the electric field *E*_*r*_ spreads out around the signal source. When the electric field arrives at the interface between PML and free space, it can be absorbed by the PML. So, the theory put forward above is correct.

## 3. Radiation Characteristic of Plasma Antenna

**Figure 3:**
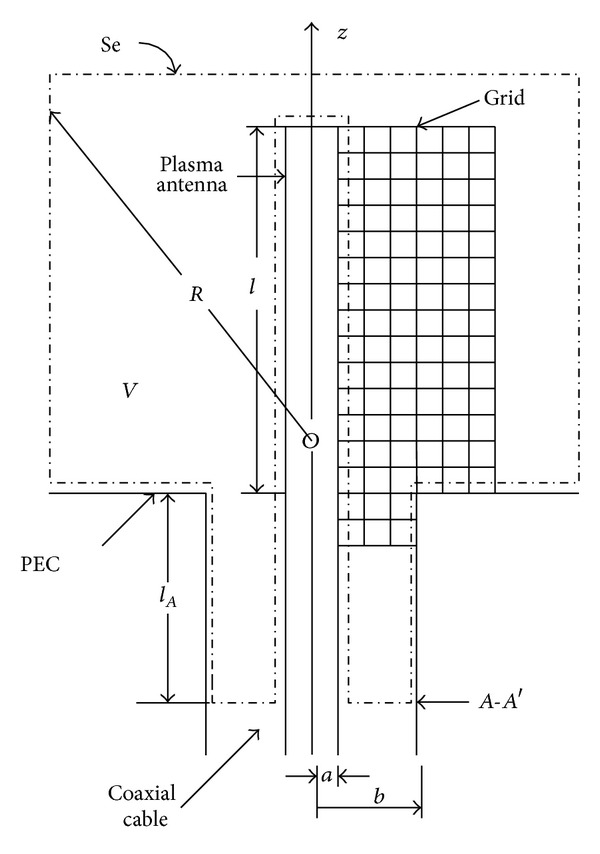
Two-dimension geometry of plasma antenna.

In this part, the radiation characteristic of plasma antenna under two-dimensional case is investigated. The geometry [17, 18] of plasma antenna is shown in Figure 3.

As Figure 3 illustrated, *V* represents free space around the plasma antenna. The plasma antenna is fed by coaxial cable. The parameters *a* and *b* are inner and outer radius of coaxial cable with the ratio of *b*/*a* = 2.3 to ensure that the characteristic impedance is 50 Ω. *l* represents the length of plasma antenna tube. By using the FDTD approach together with the theory in Section 2, we study the near-field and far-field radiation pattern of plasma antenna.

### 3.1. Near-Field Radiation Pattern

If we want to obtain the unique solution to Maxwell equation within *V*, we must initialize the electromagnetic fields **E** and **H** within *V* at time *t* = 0. Furthermore, the values **n** × **E** and **n** × **H** must be initialized also on the boundary surface for all time 0 < *t* < *t*_0_. The gauss pulse voltage source is imposed on the cross section *A*-*A*′ as shown in Figure 3. The expression of *E*_*r*_ is as follows:

(20) $$\begin{array}{c} E_{r}^{i}\left(t\right)=\frac{V^{i}\left(t\right)}{\ln\left(b/a\right)r}\hat{r}. \end{array}$$

This is the only electric field at the cross section if we choose 2*l*_*A*_ > *ct*_0_, because the field reflected from the end of the line will not reach the cross section during the observation time. The outer conductor of coaxial cable connects with ground. The inner conductor, outer conductor, and ground are considered as perfect electric conductor (PEC). So the value of **n** × **E** is zero on the surface of the coaxial cable and ground during the observation process.

**Figure 4:**
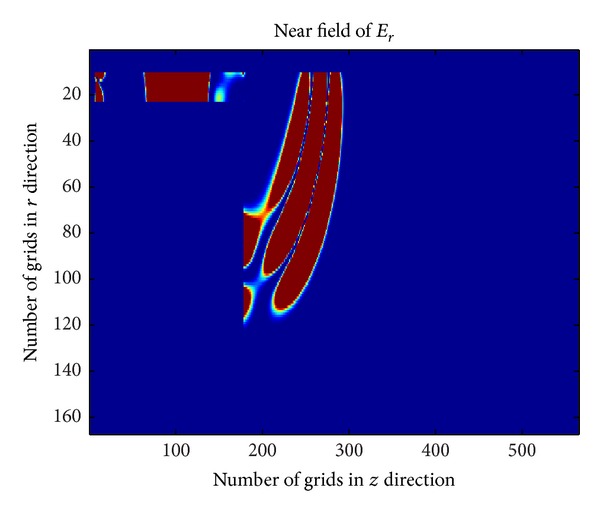
Near-field of plasma antenna with iterative number 500.

**Figure 5:**
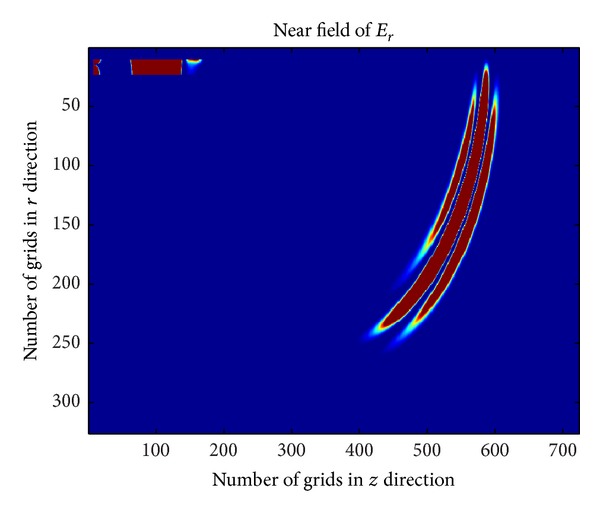
Near-field of plasma antenna with iterative number 1000.

**Figure 6:**
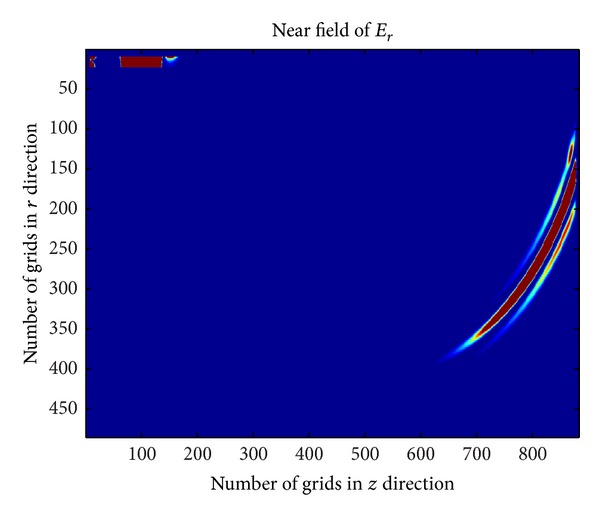
Near-field of plasma antenna with iterative number 1500.

The gauss pulse voltage source is initialized with the parameters *τ*_*a*_ = *h*/*c*, *τ*_*p*_/*τ*_*a*_ = 8 × 10^−2^. The parameters describing the plasma antenna are as follows: the length *l* = 50 cm and the radius of the conductors of the coaxial line *a* = 1 cm and *b* = 2.3 cm. The spatial step is Δ*r* = Δ*z* = (*b* − *a*)/4. The temporal step can be calculated according to the expression $\Delta t=1/c*\sqrt{1/dr^{2}+1/dz^{2}}$. Usually, the time step is chosen to be 20% smaller than the courant stability limit. The parameters of plasma are initialized: electron density is *n*_*e*_ = 1 × 10^17^ m^−3^ and collision frequency is *ν*_*c*_ = 1.5 × 10^8^ Hz. From the equation $\omega_{p}=\sqrt{e^{2}n_{e}/m\varepsilon_{0}}$, the angular frequency of plasma can be obtained as *ω*_*p*_ = 1.7815 × 10^10^ rad/s. Through FDTD method, the near-field of plasma antenna corresponding to the iterative numbers is 500, 1000, and 1500. The corresponding results are shown in Figures 4, 5, and 6.

Figure 4 ~ Figure 4 are the near-field of plasma antenna with different iterative number.  Figure 6 shows the part of the power radiated to the free space and part of power reflected back to the coaxial cable when electromagnetic wave propagates from the bottom to the joint of coaxial cable and plasma antenna.  Figure 5 shows that when the iterative number is 1000, the electromagnetic wave continues to spread out and has not reached the top of the plasma antenna. At the same time, the reverse electric field in coaxial cable will continue to propagate in signal source direction. When the iterative number comes to 1500, the electromagnetic wave will arrive at the top of the plasma antenna.  Figure 6 shows that reflection has happened and the second radiation is formed.

### 3.2. Far-Field Radiation Pattern

The finite-difference time-domain (FDTD) method [19, 20] is used to compute electric and magnetic field within a finite space around an electromagnetic object. Namely, only the value of near magnetic field can be obtained. Otherwise, we also care about the far-zone electromagnetic field of plasma antenna. The far-zone electromagnetic field can be computed from the near-field FDTD data through a near-field to far-field (NF-FF) transformation technique.

**Figure 7:**
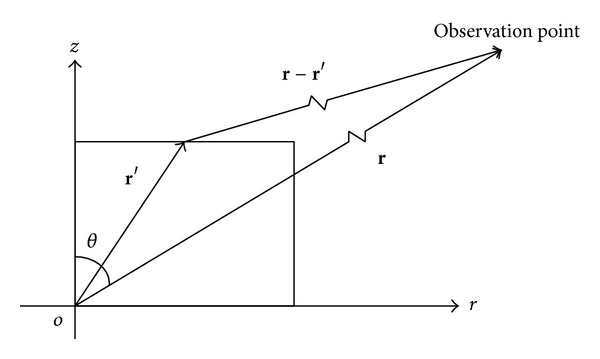
Schematic map of NF-FF transformation.

The far-field value is calculated in cylindrical coordinate. The schematic map of NF-FF is shown as in Figure 7.

The vector **r** denotes the position of the observation point (*r*, *θ*); the vector **r**′ denotes the position of source. The value of the source can be calculated through FDTD method.

Through using the Green function under two-dimension conditions, the expressions of far-zone electromagnetic field in cylindrical coordinate are

(21) Ez=exp⁡(−jkr)22jπkr(jk)(−Zfz+fmφ),Hz=exp⁡⁡(−jkr)22jπkr(−jk)(fφ+1Zfmz),

where *f*_*ζ*_(*φ*), *f*_*mζ*_(*φ*)  (*ζ* = *z*, *φ*) are current moment and magnetic moment, respectively:

(22) fζ(φ)=∫lJ(r′)exp⁡⁡(jk·r′)dl′,fmζ(φ)=∫lJm(r′)exp⁡(jk·r′)dl′.

Mapping from spherical coordinate to cylindrical coordinate, we have

(23) $$\begin{array}{c} k\cdot r^{\prime }=k\sin\left(\theta \right)\cdot r^{\prime }+k\cos\left(\theta \right)\cdot z. \end{array}$$

Substituting (23) into (22), (22) can be rewritten as

(24) fζ(φ)=∫lJζ(r′)exp⁡(j(ksin(θ)·r′+kcos⁡(θ)·z))dl′,fmζ(φ)=∫lJmζ(r′)exp⁡(j(ksin(θ)·r′+kcos⁡(θ)·z))dl′.

Substituting (24) into (21), the far-field electromagnetic field can be obtained.

Through the NF-FF method, the affection of electron density to the radiation characteristic of plasma antenna is studied. We initialize the typical parameters of plasma as below.

**Figure 8:**
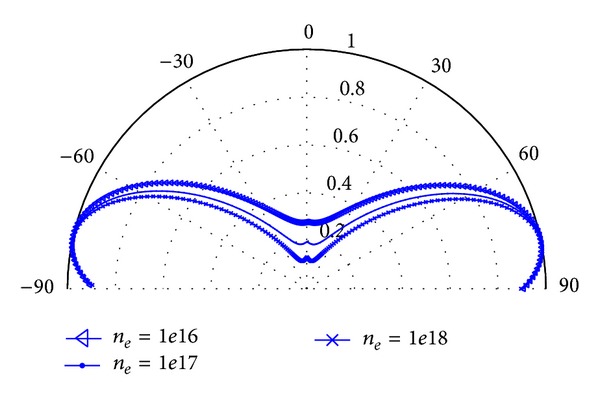
Far-field of plasma antenna under different electron density.

Collision frequency is *ν*_*c*_ = 1.5 × 10^8^ Hz, and the electron density is set as *n*_*e*_ = 1 × 10^16^ m^−3^, *n*_*e*_ = 1 × 10^17^ m^−3^, and *n*_*e*_ = 1 × 10^18^ m^−3^, respectively. And the far-field of plasma antenna under different electron density is shown as in Figure 8.

In Figure 8, it is shown that, with the variation of electron density of plasma antenna, the profile of far-field radiation pattern will change. The reason is that when the electromagnetic wave arrives at the plasma region, the interaction between electromagnetic wave and plasma changes the surface current distribution of plasma antenna, as it is known that the radiation pattern is determined by the surface current distribution of antenna. Thus, the far-field radiation pattern of plasma antenna will be changed.

## 4. Conclusion

The radiation characteristic of plasma antenna is investigated in this paper. Before studying this problem, two key issues are investigated. Firstly, we study the propagation of electromagnetic wave in free space by using FDTD method. The updating equations of Maxwell equation in stretched coordinate are derived. In order to validate the correctness of the theory, the propagation of electromagnetic wave in free space is calculated. Results show that the theory is correct and can be used in cylindrical coordinate. Secondly, the radiation characteristic of plasma antenna under two-dimension case and the near-field radiation pattern are obtained. Through the NF-FF transformation, we obtain the far-field radiation pattern. From the results, we can conclude that the electron density can influence the radiation characteristic of plasma antenna.
